# Long-Term Effects of COVID-19: Analysis of Imaging Findings in Patients Evaluated by Computed Tomography from 2020 to 2024

**DOI:** 10.3390/tomography11050049

**Published:** 2025-04-24

**Authors:** Zeynep Keskin, Mihrican Yeşildağ, Ömer Özberk, Kemal Ödev, Fatih Ateş, Bengü Özkan Bakdık, Şehriban Çağlak Kardaş

**Affiliations:** 1 Department of Radiology, Konya City Hospital, Konya 42020, Turkey; 2 Department of Chest Diseases, T.C. Ministry of Health Meram State Hospital, Konya 42090, Turkey; 3 Department of Radiology, Faculty of Medicine, Konya Chamber of Commerce Karatay University, Konya 42020, Turkey; 4 Department of Chest Diseases, Konya City Hospital, Konya 42020, Turkey

**Keywords:** COVID-19, long-term effects, computed tomography, CT severity score

## Abstract

**Background:** This study aims to systematically evaluate the findings from computed tomography (CT) examinations conducted at least three months post-diagnosis of COVID-19 in patients diagnosed between 2020 and 2024. **Objective:** To determine the frequency and characteristics of CT findings in the post-COVID-19 period, analyze long-term effects on lung parenchyma, and contribute to the development of clinical follow-up and treatment strategies based on the collected data. **Materials and Methods:** Ethical approval was obtained for this retrospective study, and individual consent was waived. A total of 76 patients were included in the study, aged 18 and older, diagnosed with COVID-19 between March 2020 and November 2024, who underwent follow-up chest CT scans at 3–6 months, 6–12 months, and/or 12 months post-diagnosis. CT images were obtained in the supine position without contrast and evaluated by two experienced radiologists using a CT severity score (CT-SS) system, which quantifies lung involvement. Statistical analyses were performed using IBM SPSS 23.0, with significance set at *p* < 0.05. **Results:** The results indicated a mean CT-SS of 10.58 ± 0.659. Significant associations were found between age, CT scores, and the necessity for intensive care or mechanical ventilation. The most common CT findings included ground-glass opacities, reticular patterns, and traction bronchiectasis, particularly increasing with age and over time. **Conclusion:** This study emphasizes the persistent alterations in lung parenchyma following COVID-19, highlighting the importance of continuous monitoring and tailored treatment strategies for affected patients to improve long-term outcomes.

## 1. Background

The COVID-19 pandemic has had profound and lasting effects on healthcare systems worldwide, posing a significant threat to human health [1]. As of 23 February 2023, more than 750 million people have recovered from COVID-19 globally; however, concerns remain regarding the potential long-term damage to certain organs, particularly the lungs, following infection [2,3]. The infection caused by the SARS-CoV-2 virus is associated not only with acute complications but also with various health issues in the post-acute phase. The lung damage caused by COVID-19 is a significant concern for patients in the long term [4]. In this context, a systematic examination of the long-term effects of COVID-19 has become a critical requirement for improving clinical practice and patient care.

The aim of this study is to systematically evaluate the findings obtained from computed tomography (CT) examinations conducted at least three months after the diagnosis of COVID-19 in patients diagnosed between 2020 and 2024. The primary objectives of the study include determining the frequency and characteristics of CT findings in the post-COVID-19 period, analyzing the long-term effects on lung parenchyma, and contributing to the development of clinical monitoring and treatment approaches based on the data obtained.

## 2. Materials and Methods

### 2.1. Study Population

Ethical approval was obtained for this study, and due to its retrospective nature, the requirement for individual consent was waived. These patients had undergone repeat chest CT scans for various reasons, with follow-up imaging conducted at least three months after the initial diagnosis. Among these, three patients had both 3–6-month and 6–12-month follow-up scans, two patients had both 6–12-month and 12-month scans, two patients had both 3–6-month scans and scans beyond 12 months, and eleven patients had scans in all three categories. The total number of cases was determined to be 111.

### 2.2. Image Analysis and Evaluation of Chest CT Scores

Chest imaging was performed using a 128-slice dual-source CT (MSCT; SOMATOM Flash Definition, Siemens, Forchheim, Germany) with automatic dose modulation software (CARE Dose4D™, Siemens, Forchheim, Germany) (Scan parameters: 200 mAS, 120 kV, 200 mm FOV). The slice thickness was 1 mm, with an increment of 0.5 mm. Images were acquired in the supine position without contrast and were evaluated at the workstation in the axial plane using lung parenchyma windows.

Chest CT scans at the time of admission and at the specified follow-up dates were evaluated by two expert radiologists with over ten years of experience, reaching a consensus. All images were retrospectively assessed through the Radiology Information System (RIS). A computed tomography severity scoring system (CT-SS) was developed to express the severity and extent of COVID-19 pneumonia more clearly, and this scoring system was utilized in our study. This system semiquantitatively indicates the degree of lung lobe involvement. Anatomically, both lungs consist of a total of five lobes and 18 segments. For standardization and symmetry, evaluations were made as if there were 20 segments (the left upper lobe apicoposterior segment was subdivided into apical and posterior segments; the left lower lobe anteromedial-basal segment was divided into anterior and mediobasal segments) [5]. CT images were assessed for the extent of involvement of specific lobes, the location of lesions, and whether the lung involvement was focal or multifocal. The patterns of lesions were classified by consensus into ground-glass opacities, consolidation, parenchymal band formation, reticular patterns, presence or absence of volume loss, traction bronchiectasis and/or bronchiolectasis, cyst presence, parenchymal distortion, mosaic attenuation, and honeycombing. Once the CT findings at diagnosis were described, the presence of at least one of these findings was considered as involvement of that segment. Each affected segment was scored as “0”, “1”, or “2” (0 points for no involvement, 1 point for involvement of less than 50% of the segment, and 2 points for involvement of more than 50%). Each of the described 20 regions of the lung was individually scored as “0”, “1”, or “2”, resulting in a CT-SS range of 0–40 [5]. After scoring, follow-up CT scans at 3–6 months, 6–12 months, and beyond 12 months were evaluated for the presence of parenchymal pattern lesions, recorded as present or absent. Lesions identified in scans beyond 12 months were recorded as permanent abnormalities.

### 2.3. Statistical Analysis

Statistical analyses were performed using IBM SPSS 23.0 (IBM Corp., Armonk, NY, USA). Numerical variables were expressed as mean ± standard deviation or count (percentage), depending on suitability. The normality of distribution for variables was assessed using visual (histogram and probability plots) and analytical methods (Kolmogorov-Smirnov and Shapiro-Wilk tests). Descriptive analyses for non-normally distributed variables were presented using the median and interquartile range. Since the CT score and age showed normal distribution, comparisons were made between these parameters and the groups based on gender, intensive care admission, and mechanical ventilation using Student’s *t*-test. Comparisons between groups for gender, intensive care admission, mechanical ventilation, and lung parenchyma patterns at 3–6 months, 6–12 months, and over 1 year were performed using the Chi-square test. Results with a *p*-value less than 0.05 were considered statistically significant.

## 3. Results

In our patients, the CT-SS score ranged from a minimum of 3 to a maximum of 27, with a mean of 10.58 ± 0.659. The age of the patients ranged from 18 to 88 years, with a mean of 51.26 ± 1.822. In the 3–6-month period, normal radiological findings were observed in 12 out of 51 cases (24%); in the 6–12-month period, normal findings were present in 9 out of 27 cases (33%); and in the periods exceeding one year, normal findings were observed in 18 out of 33 cases (55%), indicating complete radiological recovery. The CT parenchymal findings categorized by gender in patients with parenchymal findings during the 3–6 month, 6–12 month, and over 1-year periods are presented in Table 1. The key characteristics of the patient population and imaging findings are summarized in Table 2.

CT findings are presented as number of patients and percentage of the group. The final column shows the Chi-square test *p*-value assessing trends across time intervals, combining male and female counts per period. Statistically significant trends (*p* < 0.05) are marked.

This table presents a structured summary of the demographic data, comorbid conditions, COVID-19 diagnosis period, CT scan timing and indications, and categorized imaging findings of patients included in the study. The reorganization of comorbidities and temporal imaging results offers a clear understanding of both the patient population and the long-term pulmonary effects observed through CT imaging.

In the Student’s *t*-test, no significant differences were found in age and CT scores between genders. As age and the severity of the CT score increased, the rate of admission to the intensive care unit and the need for mechanical ventilation were found to be significantly higher (*p* = 0.013, *p* < 0.001).

**Figure 1:**
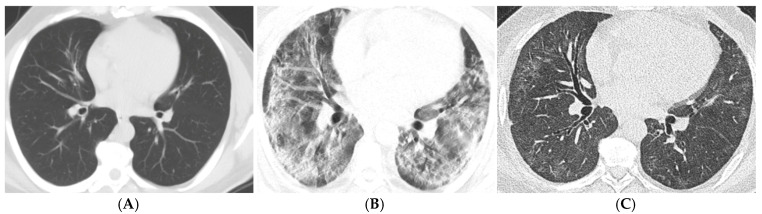
Axial CT scans of a 44-year-old male patient with moderate to severe COVID-19. (**A**) The images obtained two months prior to the onset of symptoms show that the lung parenchymal areas are completely normal, with no significant traction bronchiectasis observed. (**B**) The CT obtained 11 days after the onset of symptoms reveals ground-glass opacities, consolidation areas, and traction bronchiectasis bilaterally and in all lobes, with a CT severity score of 27. (**C**) The follow-up CT scan performed four months later shows patchy, blurry ground-glass opacities and markedly pronounced traction bronchiectasis that were not evident in prior scans before the COVID-19 infection.

In the Student’s t-test during the 3–6-month period, patients with higher CT scores had significantly higher rates of ground-glass opacities, parenchymal bands, reticular densities, traction bronchiectasis (Figure 1A–C), and mosaic attenuation (*p* = 0.003, *p* = 0.013, *p* = 0.024, *p* < 0.001, *p* = 0.025). Additionally, in the 3–6-month period, as age increased, the occurrence of parenchymal bands, traction bronchiectasis, and parenchymal distortion was significantly higher (*p* = 0.009, *p* = 0.014, *p* = 0.031).

In the Student’s *t*-test during the 6–12-month period, patients with higher CT scores had significantly higher rates of traction bronchiectasis, and as age increased, the occurrence of parenchymal bands was also significantly higher (*p* = 0.026, *p* = 0.039).

**Figure 2:**
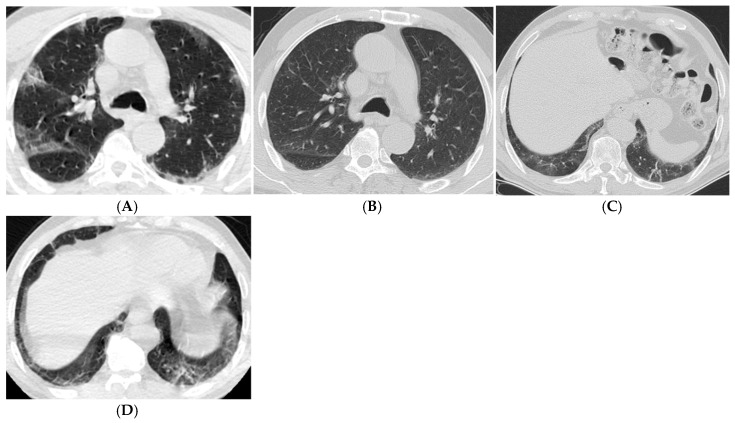
Axial CT scans of a 67-year-old male patient with moderate COVID-19. (**A**,**B**) The CT obtained 9 days after the onset of symptoms shows peripheral ground-glass opacities, consolidation areas, and curvilinear and linear fibrotic bands on both sides, with a CT severity score of 16. (**C**) The follow-up CT performed two years later reveals no significant pathological findings at the carinal level compared to the previous examination. (**D**) In the left lower lobe, more pronounced patchy, blurry ground-glass densities, thick parenchymal bands, and persistent fibrotic densities are observed in the posterior basal segments.

In the Student’s *t*-test during the period exceeding one year, patients with higher CT scores exhibited (Figure 2A–D) significantly higher rates of ground-glass opacities, reticular densities, traction bronchiectasis, mosaic attenuation, and parenchymal cysts (*p* = 0.002, *p* = 0.011, *p* < 0.001, *p* = 0.040, *p* = 0.013). Moreover, as age increased in the period exceeding one year, the occurrence of ground-glass opacities, parenchymal bands, and traction bronchiectasis was significantly higher (*p* = 0.040, *p* = 0.007, *p* = 0.046).

**Figure 3:**
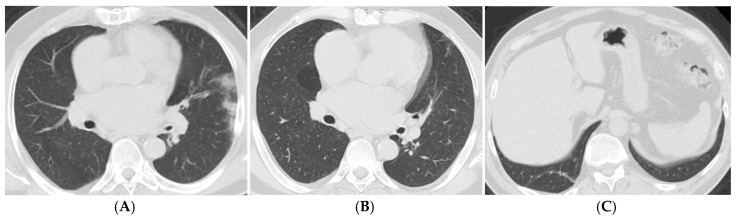
Axial CT scans of a 63-year-old male patient with mild to moderate COVID-19. (**A**) The CT obtained seven days after the onset of symptoms shows patchy, peripheral ground-glass opacities and consolidation areas bilaterally, with a CT severity score of 12. (**B**) The follow-up CT performed seven months later reveals the presence of air trapping in the medial segment of the right middle lobe, which was not observed in the previous examination. (**C**) In the right lower lobe, thick parenchymal bands and fibrotic densities are noted in the posterior basal segment.

**Figure 4:**
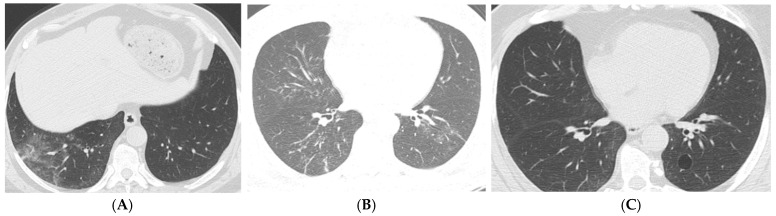
Axial CT scans of a 68-year-old male patient with mild to moderate COVID-19. The CT obtained 4 days after the onset of symptoms shows a CT severity score of 8; however, the patient was admitted to the intensive care unit and placed on mechanical ventilation 7 days later. (**A**) The scans reveal peripheral ground-glass opacities, consolidation areas, traction bronchiectasis, and bronchiolectasis in the posterior basal segment of the right lower lobe. (**B**) At the mid-zone level, linear patchy consolidation areas, mild ground-glass densities, and slight bronchiectasis are observed. (**C**) The follow-up CT performed 13 months later shows a cyst in the anterior basal segment of the left lower lobe, which was not evident in the previous examination.

In the Chi-square test during the 3–6-month period, patients admitted to the intensive care unit had significantly higher rates of reticular fibrosis, traction bronchiectasis, and parenchymal distortion (*p* = 0.017, *p* < 0.001, *p* = 0.038). Among patients receiving mechanical ventilation during the 3–6-month period, the rates of ground-glass opacities, parenchymal bands, and traction bronchiectasis were significantly higher (*p* = 0.037, *p* = 0.042, *p* = 0.013). In the 6–12-month period (Figure 3A–C), patients admitted to the intensive care unit showed significantly higher rates of parenchymal bands and parenchymal distortion (*p* = 0.009, *p* = 0.023). In the period exceeding one year, patients admitted to the intensive care unit had significantly higher rates of traction bronchiectasis, mosaic attenuation, parenchymal distortion, and parenchymal cysts (*p* = 0.003, *p* = 0.005, *p* = 0.001, *p* = 0.012) (Figure 4A–C). Among patients receiving mechanical ventilation in the period exceeding one year, the rates of traction bronchiectasis and cysts were significantly higher (*p* = 0.026, *p* = 0.038).

## 4. Discussion

The COVID-19 pandemic has had significant and lasting impacts on healthcare systems worldwide, particularly raising concerns about the risk of long-term lung damage [6,7]. Computed tomography (CT) is an effective method for diagnosing COVID-19, having detected the virus in one-third of asymptomatic patients, which is crucial in preventing transmission [8]. The role of CT in the management of COVID-19 is important for identifying both acute and chronic lasting effects. CT serves as a vital component, not only for diagnostic purposes but also for prognostic and therapeutic considerations [8]. In this study, we systematically evaluated the long-term effects on lung parenchyma through CT examinations performed after three months and later in patients diagnosed with COVID-19. The findings emphasize the potential for COVID-19 to cause permanent damage to the lungs and the necessity for monitoring such damage. Our results revealed significant relationships between CT severity scores (CT-SS) and patient demographic characteristics following COVID-19. Notably, a positive correlation was found between age and CT-SS; as age increased, so did the rates of admission to intensive care and the need for mechanical ventilation. The significantly high occurrence of parenchymal findings during the 3–6-month follow-up period suggests that the lung damage caused by COVID-19 continues even during the recovery process [5,6,7]. Similar to many studies, our findings indicate that the severity of CT findings related to COVID-19 is greater in the elderly population, largely due to the increased prevalence of severe chronic illnesses [9,10,11].

The significantly high frequency of parenchymal findings observed in the 3–6-month period indicates that patients recovering from COVID-19 may experience permanent changes in lung parenchyma. Furthermore, in the follow-up assessments exceeding one year, parenchymal damage observed in patients treated in the intensive care unit suggests that the lasting effects of COVID-19 on the lungs become more pronounced over time, with long-term repercussions on the severity of the disease and the clinical status of patients. In a large cohort from Wuhan, China, more than half of the patients scanned by CT six months after hospitalization had abnormal findings, with the most common findings being pulmonary interstitial changes such as ground-glass opacities and parenchymal bands, consistent with our study [2]. Solomon et al. [9] concluded that the CT appearances in post-acute sequelae of COVID-19 (PASC) should be standardized primarily as ground-glass opacities, predominantly fibrotic, and mixed ground-glass and fibrotic patterns. In line with our study, a cohort of hospitalized patients without mechanical ventilation showed improvements in tomographic, pulmonary function, and exercise-related variables, yet 24% still had abnormalities in CT scans one year post-discharge [6,12]. Similarly, another study found that patients experiencing significant acute symptoms of COVID-19 typically exhibited radiographic signs such as coarse subpleural reticulation without the classic signs of fibrosis, bronchial dilation, or parenchymal bands [13]. The honeycombing appearance was present in a limited number of patients (n = 4), three of whom had a diagnosis of interstitial lung disease, with one lacking previous CT scans. Among these three patients, there was significant progression of findings, and fibrosis had worsened, leading to their admission to the intensive care unit. In another study, participants with fibrotic-like changes in the lungs had a higher incidence of ARDS (25 out of 40 patients, or 63%), suggesting that such changes may serve as a predictor [14]. Previous studies [15,16] have shown that a significant portion of patients who survive ARDS may develop progressive fibrotic-like changes in their CT scans. However, it remains uncertain whether the fibrotic-like changes observed in this study truly represent actual fibrotic lung disease. The decreasing prevalence of parenchymal findings in later follow-up scans suggests the need for future research to determine whether these fibrotic-like changes reflect permanent alterations in the lungs. The presence of patients receiving mechanical ventilation in our study, along with the high occurrence of fibrotic-like changes among them, presents a risk factor. Based on previously published data [14,17], mechanical ventilation is strongly associated with the fibrotic-like changes observed after ARDS. Similarly, the increased frequency of fibrotic-like changes in the lungs of our patients, along with the fact that three-quarters of the cases with cysts in patients scanned over one year were those who had been subjected to mechanical ventilation, may also be linked to ventilator-induced lung injury. Some patients have been reported to develop cystic lung disease, such as pneumatocele. Studies have indicated that, as in our findings, these changes are often attributed to barotrauma during mechanical ventilation (ventilator-induced lung injury) [14,18,19,20]. Although radiological features of cyst formation secondary to COVID-19 have been documented, they occur with relatively low frequency [19].

In our study, a significant reduction was observed in the CT scores for total lesions, ground-glass opacities, and consolidation in follow-up CT scans compared to the initial CT. Although the predominant CT patterns in follow-up scans remained ground-glass opacities and parenchymal bands, the densities visually appeared reduced. An increasing extension of ground-glass opacities or consolidation with decreased attenuation has been reported in follow-up CT scans of COVID-19 pneumonia, indicating a gradual resolution of alveolar inflammation and re-expansion [21,22]. The underlying pathophysiology and correlation of ground-glass opacities with fibrosis during the recovery phase of COVID-19 pneumonia warrant further investigation.

We found that a higher CT-SS at the initial examination is an independent prognostic factor for the presence of fibrotic-like changes. According to a previous study on idiopathic pulmonary fibrosis [23], the CT score correlates with the degree of pulmonary fibrosis in pathological specimens. Furthermore, a recent publication has shown an association between a CT score of 18 or higher and an increased risk of mortality in COVID-19 patients [24]. Therefore, greater lung damage during the acute phase may be linked to higher mortality rates in survivors and more severe pulmonary sequelae. Additionally, utilizing a standardized CT scoring system to assess post-COVID-19 lung damage may contribute to clinical practice.

This study has several important limitations:

Observational Design: The study has a retrospective design, which can complicate the determination of cause-and-effect relationships and increase the risk of bias in the interpretation of results.

Patient Population: The study is limited to patients from a specific geographical region. Therefore, the generalizability of the findings may not apply to populations from different geographical areas or those with diverse demographic characteristics.

Inadequate Long-term Follow-up: Some patients were followed for only 12 months or a shorter duration, which restricts a comprehensive evaluation of long-term effects. Longer follow-up periods could facilitate a better understanding of the lasting impacts of COVID-19 on the lungs.

CT Examination Protocols: The CT examinations can vary across different centers, which may limit the standardization of results. The protocols used at each center can affect image quality and the accuracy of interpretations.

Radiological Evaluation: The CT findings were assessed by two expert radiologists, and the subjective nature of these evaluations, along with inter-observer variability, may affect the consistency of the findings. Standardizing radiological evaluations could enhance consistency among different observers.

Other Factors: The study did not sufficiently control for potential confounding factors such as patients’ comorbidities, lifestyle factors, and treatment processes. This oversight may lead to the exclusion of other significant variables that could influence the effects of COVID-19 on the lungs.

Short-term Follow-up: The limited number of CT assessments for some patients makes it challenging to fully analyze their recovery processes. A longer follow-up duration and more imaging data could provide a more comprehensive evaluation.

These limitations indicate that the findings of this study should be interpreted in a broader context and that the results may not be universally applicable. Future studies should be designed to address these limitations and provide more comprehensive data on the long-term effects of COVID-19.

In conclusion, the long-term effects of COVID-19 on lung health represent a significant issue for clinical practice and patient care. These findings highlight the need to strengthen follow-up processes post-COVID-19 and to develop strategies aimed at preserving lung health. Future research will contribute to enhancing the understanding in this area and optimizing the treatment and follow-up processes for patients recovering from COVID-19.

## Figures and Tables

**Table 1:** Comprehensive CT findings by gender, time since COVID-19 diagnosis, and statistical significance.

Finding	3–6 Mo Male (n = 20)	3–6 Mo Female (n = 31)	6–12 Mo Male (n = 11)	6–12 Mo Female (n = 16)	>12 Mo Male (n = 14)	>12 Mo Female (n = 19)	*p*-Value (Trend)
Ground-glass opacity	10 (50%)	22 (71%)	5 (45%)	9 (56%)	6 (43%)	4 (21%)	0.015
Parenchymal band	11 (55%)	22 (71%)	6 (55%)	12 (75%)	8 (57%)	9 (47%)	0.385
Reticular pattern	1 (5%)	9 (29%)	1 (9%)	2 (12%)	4 (29%)	3 (16%)	0.552
Traction bronchiectasis	3 (15%)	11 (35%)	3 (27%)	8 (50%)	7 (50%)	6 (32%)	0.379
Mosaic attenuation	2 (10%)	5 (16%)	2 (18%)	3 (19%)	3 (21%)	6 (32%)	0.301
Septal thickening	1 (5%)	2 (6%)	0 (0%)	0 (0%)	0 (0%)	0 (0%)	0.163
Honeycombing	0 (0%)	1 (3%)	1 (9%)	0 (0%)	1 (7%)	1 (5%)	0.616
Parenchymal distortion	2 (10%)	6 (19%)	3 (27%)	4 (25%)	3 (21%)	3 (16%)	0.542
Atrophy	0 (0%)	1 (3%)	1 (9%)	0 (0%)	1 (7%)	1 (5%)	0.616
Cyst	0 (0%)	0 (0%)	0 (0%)	0 (0%)	3 (21%)	1 (5%)	0.007

**Table 2:** Summary of key patient characteristics and CT imaging findings in post-COVID-19 follow-up.

Category	Parameter	Value
Demographics	Mean Age (years)	51.26 ± 1.82
Demographics	Gender Distribution (F/M)	44/32
Comorbidities	Hypertension	Present in 35% of patients
	Diabetes Mellitus	Present in 30% of patients
	Chronic Obstructive Pulmonary Disease (COPD)	Present in 20% of patients
	Cardiovascular Disease	Present in 18% of patients
	Obesity (BMI > 30)	Present in 25% of patients
COVID-19 Diagnosis	Mean Diagnosis Date	March 2020–November 2024
COVID-19 Diagnosis	Follow-Up Duration	3–6, 6–12, >12 months
CT Scan Info	Scan Indications	Post-COVID follow-up, respiratory symptoms
CT Scan Info	Scan Frequency per Patient	1–3 scans per patient
Imaging Findings (3–6 Months)	Common Findings	Ground-glass opacities, parenchymal bands, traction bronchiectasis
Imaging Findings (6–12 Months)	Common Findings	Parenchymal bands, traction bronchiectasis
Imaging Findings (>12 Months)	Common Findings	Persistent fibrotic changes, cysts, ground-glass opacities

## Data Availability

The datasets generated or analyzed during the study are available from the corresponding author on reasonable request.
